# A Physiological and Molecular Docking Insight on Quercetin Mediated Salinity Stress Tolerance in Chinese Flowering Cabbage and Increase in Glucosinolate Contents

**DOI:** 10.3390/plants13121698

**Published:** 2024-06-19

**Authors:** Waheed Akram, Imran Khan, Areeba Rehman, Bareera Munir, Juxian Guo, Guihua Li

**Affiliations:** 1 Guangdong Key Laboratory for New Technology Research of Vegetables, Vegetable Research Institute, Guangdong Academy of Agricultural Sciences, Guangzhou 510640, China; waheedakram.fas@pu.edu.pk (W.A.); imrankhan@gdaas.cn (I.K.); 2 Department of Plant Pathology, Faculty of Agricultural Sciences, University of the Punjab, Lahore 54000, Pakistan; 3 College of Earth and Environmental Sciences, University of the Punjab, Lahore 54590, Pakistan; areebarehman453@gmail.com (A.R.); bareeramunir@yahoo.com (B.M.)

**Keywords:** salinity, Chinese flowering cabbage, *Brassica rapa*, quercetin dihydrate, antioxidants, foliar application

## Abstract

The present study was performed to investigate the negative impact of salinity on the growth of Chinese flowering cabbage (*Brassica rapa* ssp. *chinensis* var. *parachinensis*) and the ameliorative effects of quercetin dihydrate on the plant along with the elucidation of underlying mechanisms. The tolerable NaCl stress level was initially screened for the Chinese flowering cabbage plants during a preliminary pot trial by exposing the plants to salinity levels (0, 50, 100, 150, 200, 250, 300, 350, and 400 mM) and 250 mM was adopted for further experimentation based on the findings. The greenhouse experiment was performed by adopting a completely randomized design using three different doses of quercetin dihydrate (50, 100, 150 µM) applied as a foliar treatment. The findings showed that the exposure salinity significantly reduced shoot length (46.5%), root length (21.2%), and dry biomass (32.1%) of Chinese flowering cabbage plants. Whereas, quercetin dihydrate applied at concentrations of 100, and 150 µM significantly diminished the effect of salinity stress by increasing shoot length (36.8- and 71.3%), root length (36.57- and 56.19%), dry biomass production (51.4- and 78.6%), Chl *a* (69.8- and 95.7%), Chl *b* (35.2- and 87.2%), and carotenoid contents (21.4- and 40.3%), respectively, compared to the plants cultivated in salinized conditions. The data of physiological parameters showed a significant effect of quercetin dihydrate on the activities of peroxidase, superoxide dismutase, and catalase enzymes. Interestingly, quercetin dihydrate increased the production of medicinally important glucosinolate compounds in Chinese flowering cabbage plants. Molecular docking analysis showed a strong affinity of quercetin dihydrate with three different stress-related proteins of *B. rapa* plants. Based on the findings, it could be concluded that quercetin dihydrate can increase the growth of Chinese flowering cabbage under both salinity and normal conditions, along with an increase in the medicinal quality of the plants. Further investigations are recommended as future perspectives using other abiotic stresses to declare quercetin dihydrate as an effective remedy to rescue plant growth under prevailing stress conditions.

## 1. Introduction

Chinese flowering cabbage (*B. rapa* ssp. *chinensis* var. *parachinensis*) has global economic importance and is frequently grown for foliage and seeds. The seeds can yield nutrient-rich oil that is used for cooking purposes and is a good source of biofuel.

Environmental stresses, particularly salt stress, reduce the growth, development, and production of crop plants [1]. In addition to 6% of the world’s total surface, almost 20% of irrigated agricultural land is damaged by salt [2]. The main causes of soil salinization are anthropogenic activities, including the use of saline water for irrigation, natural geological progressions, arid climates, and increased evaporation [3]. Stress brought on by salinity inhibits plant growth by excessive build-up of reactive oxygen species that cause oxidative damage in plants [4]. By 2050, more than half of the arable land may be affected by salinity, according to research warnings [5]. Furthermore, it is now crucial to use marginal regions impacted by salt effectively for agriculture [6]. If appropriate steps are not taken to reclaim the soil, salinity might become a major global environmental hazard.

The exogenous application of various synthetic environmentally friendly chemicals helps plants alleviate stress by the production of stress-related physiochemical and molecular changes. Quercetin is a flavonol phytochemical, with the potential for reducing multiple stress. It is commonly known that quercetin has an ameliorative effect on a variety of environmental stresses in both plants and animals [7,8]. Quercetin facilitates several plant physiological processes, such as seed germination, pollen growth, antioxidant machinery, and photosynthesis, as well as inducing proper plant growth and development [9]. According to Jańczak-Pieniążek, et al. [10], exogenous quercetin administration improves plant stress tolerance and boosts photosynthesis. They further observed that quercetin administration activates antioxidant machinery, and supports plant nutrition in wheat seedlings. Keilig and Ludwig-Müller [11] reported that the application of quercetin and naringenin on *Arabidopsis thaliana* ameliorated the harmful effects caused by cadmium and zinc toxicity. Likewise, Parvin et al. [12] observed the beneficial role of quercetin on tomato plants, as it enhanced the production of chlorophylls and carotenoid contents.

So far, not much work has been documented where quercetin has been used for the induction of salinity stress tolerance in plants, especially in crop plants grown under stress conditions. Similarly, limited literature is available documenting the use of quercetin as a foliar amendment on plants. Hence, the present study was designed to cover the knowledge gap regarding the use of quercetin dihydrate as a foliar amendment and its protective impacts on Chinese flowering cabbage grown under salinity stress conditions. The combined effect of salinity and exogenous elicitor (quercetin) was also seen in the production of medicinally important glucosinolate compounds in Chinese flowering cabbage plants.

## 2. Results

### 2.1. Selection of Salinity Level for Chinese Flowering Cabbage

**Figure 1:**
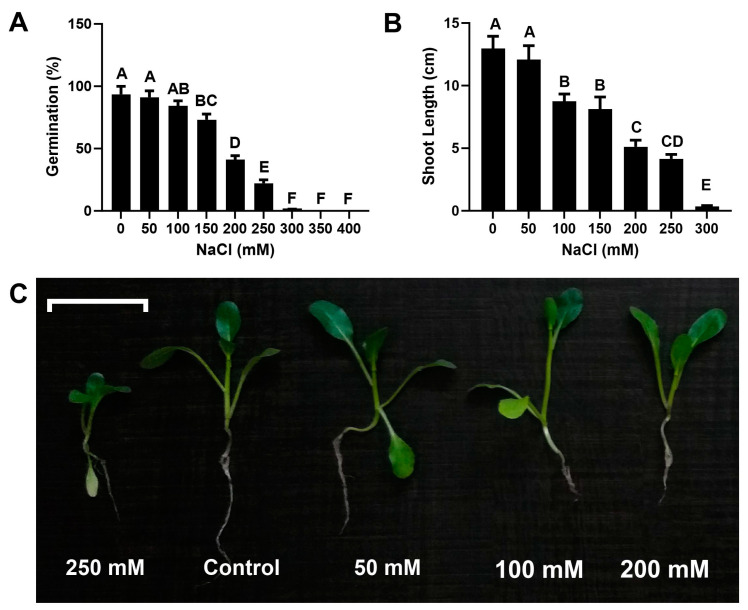
Selection of salinity level for Chinese flowering cabbage. (**A**) Response of seed germination at different salinity levels. (**B**) Shoot length of Chinese flowering cabbage at different salinity levels. (**C**) Seedling growth at different salinity levels. Capital letters on the bars show the level of significance according to ANOVA and DNMRT at *p* ≤ 0.05.

The effect of different salinity levels on germination and plant growth was used as a bioassay to select suitable salinity level/s for further experimentation. We used a range of salinity levels (0, 50, 100, 150, 200, 250, 300, 350, and 400 mM) in this pot trial. The salinity treatments caused dose-dependent germination inhibition and reduction in plant shoot length (Figure 1). Lower concentrations of 50 and 100 mM caused a non-significant reduction of 1.2% and 3.9%, respectively, in seed germination. Whereas, a relatively strong effect was seen at concentrations of >250 mM (Figure 1). Similarly, a minimum shoot length of 4.3 cm was observed at 250 mM (Figure 1). Whereas, plants failed to develop shoots when the salinity level was increased from 250 mM (Figure 1). We selected a 250 mM salinity level for the next experiments, as stronger concentrations (300 mM to 400 mM) displayed no shoot growth.

### 2.2. Ameliorative Effect of Quercetin Dihydrate on Growth Parameters of Chinese Flowering Cabbage under Salinity Stress

**Figure 2:**
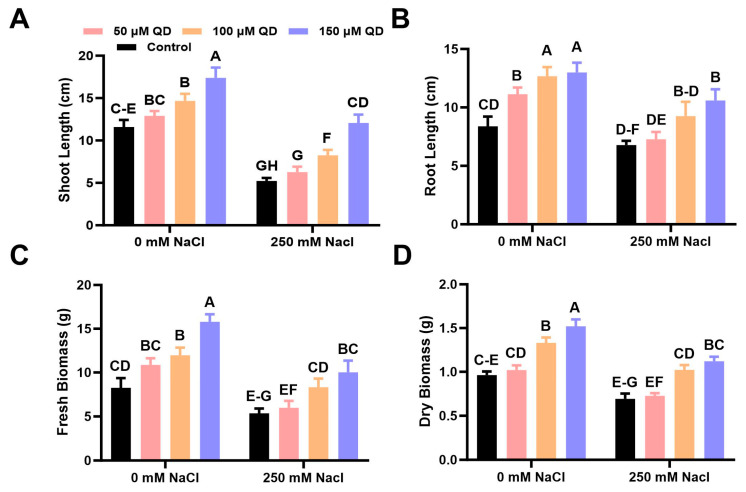
Influence of quercetin dihydrate on vegetative growth parameters of Chinese flowering cabbage under salinity stress. (**A**) Change in shoot length; (**B**) Change in root length; (**C**) Change in fresh biomass; (**D**) Change in dry biomass; QD = Quercetin dihydrate. Dissimilar letters denote significant differences among the treatments (*p* ≤ 0.05). Capital letters on the bars show level of significance according to ANOVA and DNMRT at *p* ≤ 0.05.

**Figure 3:**
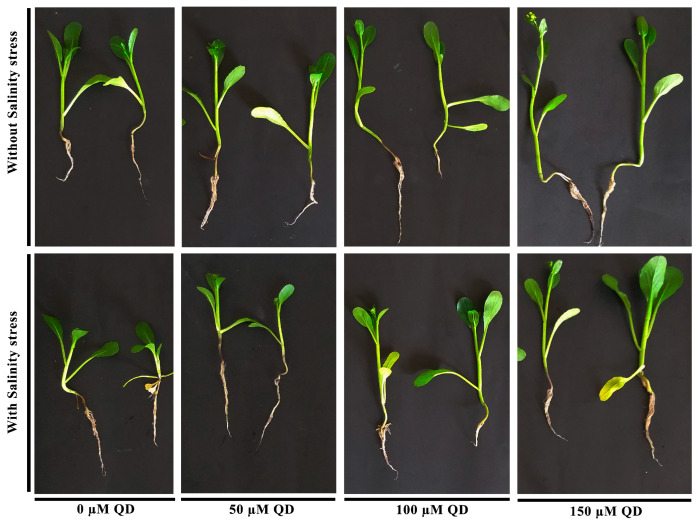
Response of Chinese flowering cabbage to foliar application of quercetin dihydrate under normal and salinity stress conditions. QD = Quercetin dihydrate. Plants were treated with quercetin dihydrate as foliar spray and salinity stress was applied as 250 mM NaCl solution as irrigation water.

Data regarding plant growth traits showed a significant positive effect on shoot length, root length, and biomass accumulation of quercetin dihydrate-sprayed plants compared to the control plants (Figure 2 and Figure 3). Under salinity stress (250 mM NaCl) conditions, the application of quercetin dihydrate at concentrations of 100 and 150 µM increased shoot length up to 36.8 and 71.3%, respectively, compared to the plants receiving salinity stress alone (Figure 2). Similarly, the lower concentration of quercetin dihydrate (50 µM) increased shoot length by up to 19.6% compared to plants receiving salinity stress alone (Figure 2). The root length was enhanced by 7.3%, 36.57%, and 56.19% under the application of 50, 100, and 150 µM quercetin dihydrate, respectively, compared to the plants receiving salinity stress alone (Figure 2B).

Additionally, fresh and dry biomasses increased significantly upon the exogenous application of quercetin dihydrate in Chinese flowering cabbage plants grown under salinity stress (250 mM NaCl) conditions. The spray of quercetin dihydrate (100 and 150 µM) significantly increased fresh biomass (51.4% and 78.6%), respectively, compared to the control plants (Figure 2C). Whereas, quercetin dihydrate significantly increased dry biomass up to 44.3% (100 µM) and 59.2% (150 µM) in saline conditions compared with the control plants (Figure 2D).

At the same time, an evident response was seen in normal conditions (Figure 2 and Figure 3). Quercetin dihydrate (100 and 150 µM) significantly increased shoot length (41.7 and 61.5%) and root length (36.9 and 52.3%), respectively, compared to the control. Under normal conditions, the application of quercetin dihydrate (150 µM) significantly increased fresh (69%) and dry biomass (62%) as compared to control (Figure 2). Quercetin dihydrate at a lower concentration (50 µM) did not significantly increase all the growth parameters.

### 2.3. Effect of Quercetin Dihydrate on Chlorophyll, Total Soluble Sugars, and Carotenoid Contents of Chinese Flowering Cabbage under Salinity Stress

The ameliorative effects of quercetin dihydrate application improved the biosynthesis of Chlorophyll, total soluble sugars (TTS), and carotenoid contents in brassica plants when raised in normal and saline circumstances (Table 1). Salinity caused a significant diminution in the mentioned biochemicals compared to normal control plants. Significantly increased biosynthesis of Chlorophyll *a* (chl *a*) and Chlorophyll *b* (chl *b*) were noted in quercetin dihydrate-treated brassica plants compared to control plants under both saline and normal conditions (Table 1). Under salinity stress (250 mM NaCl) conditions, the application of quercetin dihydrate (150 µM) increased chl *a* and chl *b* up to 2.1, 1.9-fold, respectively, compared to the control plants. Whereas, under normal conditions, the application of quercetin dihydrate (150 µM) increased chl *a* and chl *b* up to 1.6- and 1.4-fold, respectively, compared with non-treated control plants (Table 1).

The application of quercetin dihydrate significantly increased total soluble sugar (TTS) and carotenoid contents under both saline and non-saline conditions (Table 1). Under salinity stress, quercetin dihydrate at 150 µM increased TTS and carotenoid contents up to 87.0% and 41.4%, respectively, compared to the plants receiving salinity stress alone (Table 1). Similarly, under normal conditions, the application of quercetin dihydrate at 150 µM increased TTS and carotenoid contents up to 86.4% and 49.6% compared to the non-treated control plants (Table 1).

### 2.4. Effect of Quercetin Dihydrate on Antioxidant Enzyme Activities and Proline Contents of Chinese Flowering Cabbage under Salinity Stress

**Figure 4:**
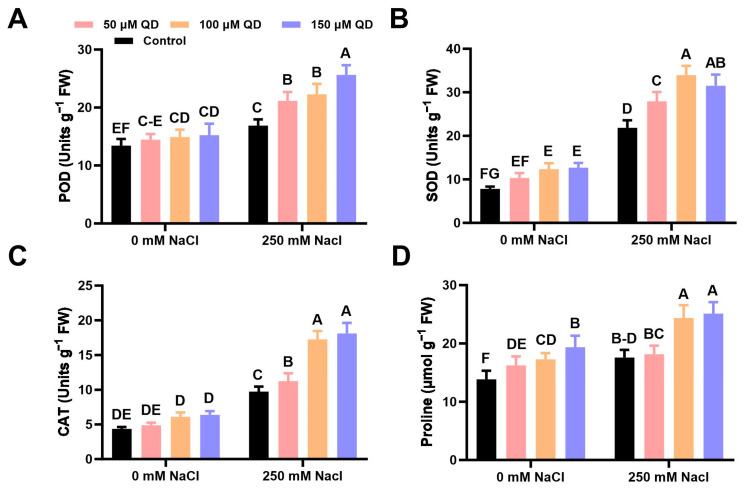
Effect of quercetin dihydrate and salinity stress antioxidative enzyme machinery of Chinese flowering cabbage plants. (**A**) Change in peroxidase (POD) activity; (**B**) Change in superoxide dismutase (SOD) activity; (**C**) Change in catalase (CAT) activity; (**D**) Change in proline contents. Data displayed as means ± standard error as shown by vertical bars. Dissimilar letters denote significant differences among the treatments (*p* ≤ 0.05).

To further investigate the mechanisms of quercetin on salinity tolerance in brassica plants, the antioxidant enzyme system was analyzed. Enzymes related to early salt stress, such as peroxidase (POD), superoxide dismutase (SOD), and catalase (CAT), were quantitatively observed. Globally, relatively higher activities of these enzymes were seen in saline-treated plants than in normal plants. As in the case of POD, higher activity was noted in saline-treated plants (12.7 units g^−1^ FW) compared to non-treated control (16.3 units g^−1^ FW) plants (Figure 4). The application of quercetin dihydrate alleviated POD activity in a dose-dependent manner (Figure 4). POD activity was increased significantly up to 1.9-fold in quercetin dihydrate (150 µM) supplied plants under saline conditions compared to the plants receiving salinity stress alone (Figure 4).

Regarding SOD, quercetin dihydrate application along with the salinity stress increased the SOD activity. The quercetin dihydrate application at 100 µM (36.2 units g^−1^ FW) showed significantly higher SOD enzyme activity when compared with the control (21.7 units g^−1^ FW) in salinity stress (Figure 4). Saline stress also increased the activity of the CAT enzyme. Yet, 150 µM quercetin dihydrate spray along with the salinity stress shows maximum CAT enzyme activity (17.4 units g^−1^ FW) (Figure 4). Likewise, CAT activity was significantly lower in brassica plants under normal conditions (4.7 units g^−1^ FW) compared to the plants under salinity stress alone (9.7 units g^−1^ FW) (Figure 4). The onset of saline stress increased the accumulation of proline (16.2 µ mol g^−1^ FW) in brassica plants compared to non-treated control (13.1 µ mol g^−1^ FW) plants (Figure 4). Quercetin dihydrate application further increased the biosynthesis of proline. Under salinity stress, the treatment of quercetin dihydrate with 100 and 150 µM increased proline contents up to 37.4% and 44.9% under saline stress compared to the plants receiving salinity stress alone (Figure 4).

### 2.5. Effect of Quercetin Dihydrate on Glucosinolate Contents of Chinese Flowering Cabbage under Salinity Stress

**Figure 5:**
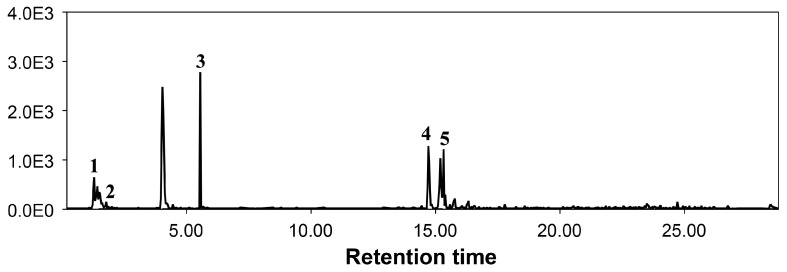
Representative chromatogram of glucosinolates detected in Chinese flowering cabbage. 1 = Progoitrin, 2 = Glucoalyssin, 3 = Gluconapin, 4 = Neoglucobrasscin, and 5 = Glucobrassicanapin.

Glucosinolate contents were quantified using liquid chromatography (Figure 5). Table 2 shows the effect of salinity and quercetin dihydrate on the quantities of glucosinolates in brassica plants. The changes were seen in both indole and aliphatic glucosinolates with varying trends. Application of salinity stress (250 mM NaCl) reduced quantities of Progoitrin, Gluconapin, Glucobrassicanapin, and 4-Hydroxyglucobrassicin up to 2.43-, 1.58-, 2.5-, and 1.5-fold, respectively, compared to non-treated control plants (Table 2). Under salinity stress, the application of 150 µM of quercetin dihydrate increased Progoitrin, Gluconapin, and 4-Hydroxyglucobrassicin up to 1.89-, 1.71-, and 1.83-fold respectively, compared to plants raised under salinity stress alone (Table 2). The quercetin dihydrate showed strong increments in the different glucosinolate contents under normal conditions. Here, the application of 150 µM of quercetin dihydrate increased Progoitrin, Gluconapin, and 4-Hydroxyglucobrassicin up to 1.29-, 1.03-, and 1.35-fold respectively, compared to non-treated control plants (Table 2).

### 2.6. Molecular Docking Analysis

**Figure 6:**
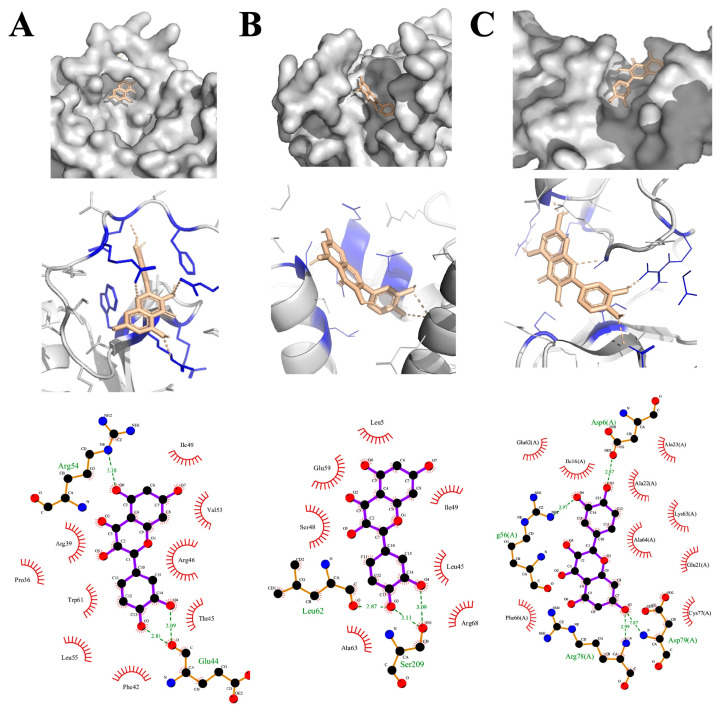
Molecular docking and interaction analysis of quercetin legend with different stress-responsive proteins of Chinese flowering cabbage. (**A**) DREB; (**B**) SOS3 and (**C**) STXBP1.

Three stress-responsive proteins, Dehydration-responsive element-binding protein (DREB), Salt overly sensitive (SOS3), and syntaxin binding protein 1 (STXBP1), were used for the molecular docking analysis (Figure 6). A molecular docking study showed binding affinity ranging from −6.9 to −8.1 three stress-responsive proteins of *B. rapa* (Table 3). DREB1 protein exhibited a binding affinity score of −7.3 with quercetin dihydrate. Whereas, SOS3 and STRP proteins were docked with the quercetin dihydrate with binding affinities of −6.9 and −8.1, respectively (Table 3). Additionally, in all three complexes between quercetin dihydrate and stress-responsive > 3 hydrogen bonding was seen, further confirming the strong bonding affinities. Details of the interactions between proteins and quercetin dihydrate are presented in Figure 6 and Table 3.

## 3. Discussion

Plants are generally more vulnerable to environmental stresses because of their sedentary existence [13]. In the preliminary experiment, Chinese flowering cabbage plants were exposed to varying levels of salinity stress (0 mM to 400 mM NaCl) provided as irrigation water. This experiment was intended to choose suitable concentrations, ensuring maximum damage along with the viability of plants for subsequent experimentation. The relationship between salinity and the germination rate was almost linear. This can be explained as the salinity stress negatively affecting the ability of the seed to absorb water, hence preventing seed germination [14]. The plants subjected to salt stress showed a significant drop in plant growth. There were concentration-dependent differences in the responses against different doses of NaCl in Chinese flowering cabbage plants. These findings correlate with those of Yildirim, et al. [15]. They demonstrated that salt significantly reduced the growth characteristics of squash plants. Inadequate photosynthesis decreases stomatal conductance and reduced carbon dioxide uptake [16,17] may be contributing factors to the lowering of the growth rate of Chinese flowering cabbage plants under stress in our study.

Considering the limits of the biological potential of crop plants, it makes sense to look for additional compounds that can lessen environmental stress and boost production. These compounds, also referred to as elicitors/stimulants, help boost plant physiological activity by promoting the synthesis of proteins [17]. These chemicals are gaining importance in agriculture because they modulate plant responses that make them resistant to adverse environmental circumstances [18]. As was observed in this study, brassica plants can withstand abiotic stressors because of the exogenous application of quercetin dihydrate. Our findings indicate that the foliar application of quercetin dihydrate was crucial in enhancing the growth of Chinese flowering cabbage plants under salt stress and raising biomass production. Under both saline stress and non-saline conditions, a significant increase in the growth parameters of the plants was observed. Application of quercetin dihydrate increased shoot length, root length, and biomass accumulation in Chinese flowering cabbage plants. A possible explanation could be the fact that quercetin dihydrate can increase the ability of cells to resist oxidative stress and maintain normal physiological function [19].

The amount of chlorophyll in a plant is an effective indicator of its abiotic tolerance [20]. Lower levels of chlorophyll are caused by increased activity of enzymes that break down chlorophyll and the suppression of chlorophyll biosynthesis due to increased ethylene production during salt stress [21,22]. A similar trend was observed in our study as salinity stress decreased total chlorophyll and carotenoid content in plants. Whereas, quercetin dihydrate ameliorated the decreased quantities of total chlorophyll and carotenoid content. These findings align well with prior studies, illustrating the beneficial impact of the foliar application of certain chemicals on plants. The stimulating impact of the exogenous administration of quercetin derivative was seen in the concentration of chlorophyll in tomato seedlings [12]. Quercetin improves electron transport by participating in photosynthesis [23]. Quercetin can also change the photosynthetic rate by altering the thylakoid membranes for increased light scattering [24]. This structural change can lead to an increased transfer of energy between photosystems, ultimately improving the process of photosynthesis [25]. This would have contributed to the increased chlorophyll contents in the Chinese flowering cabbage plants under saline stress.

Reactive oxygen species are harmful to plants, as these can penetrate cell membranes and reach areas of the cell that are distant from the site of production. Plants utilize antioxidant machinery to detoxify reactive oxygen species to adapt to stress conditions. It has been reported that quercetin regulates physiological processes in plants including the antioxidant system [26]. The application of quercetin dihydrate increased the activity of antioxidant enzymes including SOD, CAT, and POD. The combined interconnected action of these enzymes mitigates the harmful effects of reactive oxygen species produced inside plants in response to stress conditions [27]. As we observed, boosted activity of antioxidant-related enzymes would have contributed to withstand stress conditions by Chinese flowering cabbage plants. This is consistent with the results of Egedigwe and Udengwu [28], who found that exogenous treatment with the β-carotene improved the capacity of *Amaranthus hybridus* to scavenge reactive oxygen entities.

The prevalence of stressful conditions in plants has been linked to the re-modulation of the biosynthesis of glucosinolate (GLs) compounds in brassica plants. Previous research on different Brassicas, including canola and radish, showed variations in GLs concentrations under salinity [29]. The production of different GLs in the leaves of broccoli plants was negatively affected when broccoli plants were treated with 40 or 80 mM NaCl [30]. The same was observed in our study, as the salinity stress significantly decreased GLs in plants. Salinity stress causes tissue damage, due to which glucosinolates are released from vacuoles and rapidly hydrolyzed to glucose and other unstable intermediates [31].

The application of quercetin dihydrate increased the production of GLs under both saline and normal conditions. GLs has long been admired for multiple roles in plant defense against biotic and abiotic stresses. It has also been reported in previous research work that the increased production of glucosinolates in plants raised under salinity could protect plants from abiotic stress. del Carmen Martínez-Ballesta, et al. [32], stated that increased levels of glucosinolates under saline stress conditions could aid osmotic adjustment, thus helping to maintain cell turgor under salinity stress conditions. Considering the beneficial role of GLs in plant’s adaptation to stress, it can be hypothesized that increased production of GLs in the plants of Chinese flowering cabbage helped to ameliorate the salinity stress.

Molecular docking is a computational approach for predicting how different compounds would interact with a receptor protein. Molecular docking methods have recently been used to forecast potential interactions between small molecules and several target proteins found in plants [33]. It was deemed necessary to perform a molecular docking analysis between quercetin dihydrate and different stress-responsive proteins of *Brassica rapa* plants. Three proteins, namely dehydration-responsive element-binding protein, salt overly sensitive 3 protein, and syntaxin binding protein 1, were selected as receptors in molecular docking studies. Dehydration-responsive element-binding protein (DREB) and syntaxin binding protein 1 (STXBP1) are essential for normal plant growth and play a key role in abiotic stress management in plants [34]. Similarly, salt overly sensitive 3 (SOS3) maintains the integrity of the cytoskeleton under stress [35]. The molecular docking analysis showed a strong binding affinity between quercetin dihydrate and these three stress-responsive proteins. Additionally, hydrogen bonding was seen in all three cases of docking analysis attributable to the greater affinity of quercetin dihydrate with stress-responsive target proteins [36]. The binding of quercetin dihydrate in the active site of these proteins can interfere with their activities, thus modulating the downstream process to ameliorate the effect of salinity stress in Chinese flowering cabbage plants.

## 4. Materials and Methods

### 4.1. Selection of Tolerable Salinity Level for Chinese Flowering Cabbage

Chinese flowering cabbage plants were used to select the optimum salinity stress level for further experiments. The seeds were sterilized by washing in 2% sodium hypochlorite for five minutes. Afterward, the seeds were washed several times with distilled sterilized water. The commercially available peat moss substrate sold by Rekyva company (Šiauliai, Lithuania) was used in all the experiments. This peat moss substrate is prebuffered with NPK + Micro Nutrients. The substrate was autoclaved, dried, and passed by a 2-mm mash. The sterilized peat moss substrate was used in the seedling trays made up of plastic with 98 seedling compartments. A single seed was sown in each compartment. Seedling trays were watered with the NaCl solution (0, 50, 100, 150, 200, 250, 300, 350, and 400 mM) by adding an equal quantity of respective NaCl solution in water-holding trays. The seedlings were grown for 20 days. Data regarding the percentage germination and shoot length were noted to select tolerable salinity levels.

### 4.2. Foliar Application of Quercetin Dihydrate to Manage Salinity Stress in Chinese Flowering Cabbage Plants

The experiment was performed in a polyethylene sheet greenhouse. Plastic pots of ten-inch diameter were filled with the sterilized autoclaved peat potting mix. Five seeds were sown in the pots and left for incubation. After one week of emergence, thinning was performed and three uniform seedlings were left in each pot. Pots were kept in a completely randomized design. The allotted pots were irrigated each time with the NaCl solution (250 mM), starting after two weeks of germination. The stock solution of quercetin dihydrate was prepared in water using dimethylsulfoxide (DMSO) to enhance its solubility. Test concentrations of quercetin dihydrate (50, 100, 150 µM) were prepared using the stock solution, and the concentration of DMSO was left at 0.09% to 0.1% in the working solution. The solutions were sprayed twice at one-week intervals starting from the second week after the emergence as per the experiment design. The control plants were sprayed with the 0.1% DMSO solution. Spraying was done carefully to properly wet the leaves and avoid mixing them with the roots. After forty days of cultivation, the plants were carefully uprooted to record the growth-related data and for further downstream analysis. Ten replicate plants were included in each treatment, and the whole experiment was performed twice.

### 4.3. Estimation of Chlorophyll, Total Soluble Sugars, Carotenoid, and Proline Contents

Five plants from each treatment were randomly selected for the quantification of chlorophyll, total soluble sugars, carotenoid, and proline contents. Briefly, the chlorophyll and carotenoid contents were extracted in ethanol (95%) from fresh leaf samples, and quantifications were performed observing optical density (OD) at 470, 646 nm, and 663 nm, as mentioned by Sartory and Grobbelaar [37]. The following formulas were used to express the quantification of chl *a*, chl *b*, and carotenoid contents. Chlorophyll and carotenoid contents were expressed as mg g^−1^ fresh weight (FW) and µg g^−1^ FW, respectively.

Chl *a* = (12.21 × OD663 − 2.81 × OD646)/(1000 × W) × V

Chl *b* = (20.13 × OD646 − 5.03 × OD663)/(1000 × W) × V

Carotenoid = (1000 × OD470−3.27 × Cchl *a* − 104 × Cchl *b*)/(229 × 1000 × W) × V

Here “V” = volume of the extract in mL and “W” = fresh weight of the leaf sample in grams.

The plant material was extracted in 80% ethanol to assess the total soluble sugars (TSS) using the anthrone reagent. Following Franscistt et al.s’ [38] instructions, the absorbance from the solution was measured at 630 nm, and TSS contents were expressed as mg g^−1^ FW. For quantification of proline contents, leaf samples were extracted in 10 mL of 3% aqueous sulfosalicylic acid and reacted with ninhydrin acid and glacial acetic acid, suggested by Franscistt, David, and Robert [38]. The chromophore was extracted using Toluene and OD was observed at 520 nm. A standard curve was drawn by pure proline and expressed as micromole per gram of fresh weight.

### 4.4. Estimation of the Activities of Antioxidant Enzymes

Three antioxidant system enzymes, namely peroxidase (POD), superoxide dismutase (SOD), and catalase (CAT), were quantified. For that purpose, the plant material (2 g) was ground to a fine powder using liquid nitrogen and extracted with 20 mL of 0.1 M sodium phosphate buffer extraction buffer (pH 7.8). SOD activity was measured by the nitro blue tetrazolium reduction method [39]. The reaction mixture (6 mL) contained 2 mM riboflavin (0.2 mL), nitro blue tetrazolium (75 µM), methionine (13.33 mM), enzyme extract (0.2 mL), EDTA (0.1 mM), sodium carbonate (50 mM), and phosphate buffer (50 mM). The OD of the reaction mixture was analyzed at 560 nm. The CAT activity was analyzed using the H_2_O_2_ method [40]. The reaction mixture contained 100 µL of diluted enzymatic extract, 1 mL H_2_O_2_ (75 mM), and 3 mL phosphate buffer (0.1 M, pH 7). The OD was measured at 240 nm. Guaiacol was used as a substrate to quantify POD activity [41]. A 6 mL reaction mixture contained 0.1 M H_2_O_2_, 0.25% guaiacol, and 0.2 mL enzyme extract in 10 mM sodium phosphate (pH 7.4). The OD was measured at 470 mm. Enzyme activities were presented as units per gram of fresh weight, and one enzyme unit was defined as an absorbance change of 0.01 per minute.

### 4.5. Quantification of Glucosinolate Contents

We used a Shimadzu Ultra-Fast Liquid Chromatography (Shimadzu, Kyoto, Japan) connected with API 4000 QTrap mass spectrometer equipped with a Turbo Ion Spray probe (AB Sciex; Foster City, CA, USA) to quantify the different glucosinolates using multiple reaction monitoring mode (Table S1). For that purpose, leaves from five random plants were removed and pooled together. Extraction of glucosinolates was performed using Alkaline hydrolysis [42]. During chromatographic separations, the mobile phase A was trifluoroacetic acid (0.1%) and B was acetonitrile/trifluoroacetic acid (0.1). The details of the gradient elution program can be seen in [43]. The mass spectrometer data were acquired in the negative ionization mode. Mass spectrometry parameters, such as collision energy, declustering potential, and entrance potential, were determined using a pure sinigrin compound. The whole apparatus was controlled by the Analyst software version 1.4. The obtained data were processed on MzMine software (Version 2.5) for qualitative and quantitative analysis.

### 4.6. Molecular Docking Analysis of Quercetin Dihydrate with Stress-Responsive Proteins

The protein models of three stress-related proteins (DREB, SOS3, STXBP1) of *B. rapa* obtained from homology modeling [44] were used in this study. These were used for the molecular docking analysis in this study. The models were further refined by ModRefiner available at http://zhanglab.ccmb.med.umich.edu (accessed on 5 October 2022). The quality evaluation of the predicted 3D structure of the target proteins can be seen in our previous study [44]. The structure of the quercetin dihydrate was obtained from the NCBI PubChem compounds database. The molecular docking was performed using AMDock (version 1.5) workflow [45] that involves the preparation of receptor and ligand structures, energy minimization, active site finding, and molecular docking by AutoDock vina (version 1.1.2).

PyMOL software (version 2) was used for the necessary molecular visualization [46]. The interaction analysis of the best-docked poses was performed using LigPlot software (version 2.2) [47].

### 4.7. Statistical Analysis

All the experiments were repeated and mean data were presented. The data were statistically evaluated by performing ANOVA and DNMRT (*p* ≤ 0.05) using Excell (version 2019) add-in DSAASTAT [48].

## 5. Conclusions

Our results indicate that exposure to salinity can severely decrease vegetative growth and the production of medicinally important glucosinolate contents in Chinese flowering cabbage plants. Treating Chinese flowering cabbage plants with quercetin dihydrate can effectively ameliorate the harmful effects of salt stress on plant growth. Quercetin dihydrate treatment with 100−150 µM can significantly increase plant growth parameters along with the production of medicinally important glucosinolate compounds. By activating the antioxidant machinery, quercetin dihydrate can reduce oxidative damage in the plants. The use of this environmentally friendly, non-toxic synthetic flavanol chemical can protect plants from abiotic stress. Further studies must be performed using other crop plants and the development of a stable formulation of quercetin dihydrate which can be used in our conventional agricultural system.

## Figures and Tables

**Table 1:** Effect of quercetin dihydrate and saline stress on photosynthetic pigments, TSS, and carotenoid contents of Chinese flowering cabbage.

Treatments	Chlorophyll *a* (mg g^−1^ FW)	Chlorophyll *b* (mg g^−1^ FW)	TSS (mg g^−1^ FW)	Carotenoids Content (µg g^−1^ FW)
Control	1.27 ± 0.01 ^b^	0.56 ± 0.04 ^cd^	1.67 ± 0.13 ^c–e^	0.58 ± 0.04 ^bc^
SS	0.63 ± 0.04 ^de^	0.34 ± 0.02 ^e–g^	1.08 ± 0.08 ^fg^	0.42 ± 0.02 ^e^
QD (50 µM)	1.34 ± 0.21 ^b^	0.57 ± 0.03 ^c^	2.84 ± 0.16 ^ab^	0.66 ± 0.04 ^b^
QD (100 µM)	2.08 ± 0.08 ^a^	0.59 ± 0.04 ^c^	2.96 ± 0.17 ^a^	0.69 ± 0.05 ^b^
QD (150 µM)	2.13 ± 0.14 ^a^	0.82 ± 0.06 ^a^	3.13 ± 0.29 ^a^	0.87 ± 0.07 ^a^
QD (50 µM) + SS	0.84 ± 0.05 ^b–d^	0.39 ± 0.02 ^ef^	1.21 ± 0.07 ^f^	0.44 ± 0.02 ^e^
QD (100 µM) + SS	1.07 ± 0.12 ^bc^	0.46 ± 0.03 ^e^	1.82 ± 0.13 ^cd^	0.51 ± 0.03 ^cd^
QD (150 µM) + SS	1.36 ± 0.16 ^b^	0.68 ± 0.05 ^b^	2.02 ± 0.11 ^c^	0.59 ± 0.03 ^bc^

Data display means ± standard error. Dissimilar letters denote significant differences among the treatments (*p* ≤ 0.05). SS = Salinity stress (250 mM NaCl). QD = Quercetin dihydrate applied as a foliar spray.

**Table 2:** Effect of Quercetin dihydrate and salinity stress on the glucosinolate content of Chinese flowering cabbage.

Treatments	PRO	GLU	GLB	4HG	Others
Control	1.17 ± 0.06 ^b^	3.68 ± 0.17 ^cd^	0.74 ± 0.04 ^de^	0.034 ± 0.002 ^de^	1.06 ± 0.08 ^bc^
SS	0.48 ± 0.02 ^de^	2.32 ± 0.15 ^e–g^	0.49 ± 0.001 ^f^	0.022 ± 0.002 ^e^	0.69 ± 0.05 ^de^
QD (50 µM)	1.32 ± 0.12 ^b^	3.97 ± 0.20 ^c^	0.82 ± 0.09 ^cd^	0.037 ± 0.001 ^b–d^	1.13 ± 0.11 ^ab^
QD (100 µM)	1.51 ± 0.09 ^a^	4.54 ± 0.27 ^c^	1.28 ± 0.12 ^b^	0.042 ± 0.003 ^b^	1.18 ± 0.07 ^ab^
QD (150 µM)	1.52 ± 0.13 ^a^	4.82 ± 0.26 ^a^	1.66 ± 0.17 ^a^	0.046 ± 0.002 ^a^	1.24 ± 0.13 ^a^
QD (50 µM) + SS	0.49 ± 0.05 ^b–d^	4.11 ± 0.33 ^ef^	0.57 ± 0.08 ^f^	0.032 ± 0.001 ^de^	0.77 ± 0.04 ^cd^
QD (100 µM) + SS	0.52 ± 0.04 ^bc^	4.49 ± 0.29 ^e^	1.04 ± 0.18 ^c^	0.037 ± 0.003 ^b–d^	0.82 ± 0.06 ^b–d^
QD (150 µM) + SS	0.88 ± 0.06 ^b^	4.04 ± 0.32 ^b^	1.33 ± 0.16 ^b^	0.041 ± 0.005 ^bc^	0.94 ± 0.05 ^bc^

Data display means ± standard error. Dissimilar letters denote significant differences among the treatments (*p* ≤ 0.05). SS = Salinity stress (250 mM NaCl). QD = Quercetin dihydrate applied as foliar spray. PRO = Progoitrin; GLU = Gluconapin; GLB = Glucobrassicanapin; 4HG = 4-methoxyglucobrassicin. Glucosinolates in the “others” category include glucobrassicin, glucoalyssin, 4-hydroxy glucobrassicin, sinigrin, and neoglucobrasscin. Quantifications are provided as (μmol sinigrin equivalent g^−1^ dry biomass).

**Table 3:** Molecular docking scores and interaction analysis between quercetin dihydrate and stress-responsive proteins of Chinese flowering cabbage.

Protein	Vina Score	Hydrogen Bonds	Hydrophobic Interaction	Interacting Residues
DREB1	−7.3	3	3	ARG39, GLU44, ARG54, PHE42, LEU55, TRP61
SOS3	−6.9	4	3	SER48, LEU62, SER209, LEU45, LEU62
STRP	−8.1	4	3	ILE16, LYS63, ARG78, ALA23, ARG56, ASP79

## Data Availability

Data is contained within the article or Supplementary Material.

## Supplementary Materials

The following supporting information can be downloaded at: https://www.mdpi.com/article/10.3390/plants13121698/s1, Table S1: Parameters of glucosinolates quantification.
