# Supplementary material for: A Physiological and Molecular Docking Insight on Quercetin Mediated Salinity Stress Tolerance in Chinese Flowering Cabbage and Increase in Glucosinolate Contents
Source: Plants (Basel). 2024 Jun 19;13(12):1698. doi: 10.3390/plants13121698 (PMC11207431; doi:10.3390/plants13121698)
Supplement: Supplementary file 1 [file plants-13-01698-s001.zip › plants-3036875-supplementary.pdf]

Table S1. Parameters of glucosinolates quantification.

Compound	Precursor ion	Product ion
Glucoalyssin	450	172, 205, 291, 259
Gluconapin	372	130, 139, 179, 275, 371
Glucobrassicin	446	97, 172, 205, 259
Neoglucobrassin	477	97, 259, 275, 383, 466
Progoitrin	388	136, 146, 259, 275
4-Hydroxyglucobrassicin	463	132, 169, 221, 383, 267
4-Methoxyglucobrassicin	477	97, 137, 202, 235, 259
Sinigrin	358	97, 132, 185, 195
